# Exploring lead free Rb_2_AlInX_6_ halide double perovskites for advanced energy harvesting applications

**DOI:** 10.1039/d5ra06712j

**Published:** 2025-11-12

**Authors:** Syed Muhammad Kazim Abbas Naqvi, Sahar Abdalla, Kiran Akhtar, Aleesha Ali, Nuha Y. Elamin, Faheem Abbas, Abdul Munam Khan, Rasheed Ahmad Khera

**Affiliations:** a Faculty of Materials Science, Shenzhen MSU-BIT University Shenzhen 518115 China; b Chemistry Department, College of Science, Imam Mohammad Ibn Saud Islamic University (IMSIU) Riyadh 11623 Saudi Arabia; c Institute of Botany, University of the Punjab Lahore 54590 Pakistan; d Chemical Engineering Research Center, School of Chemical Engineering and Technology, Tianjin University Tianjin China; e Department of Chemistry, Key Lab of Organic Optoelectronics and Molecular Engineering of Ministry of Education, Tsinghua University Beijing 100084 PR China; f Department of Physics and Microelectronics, Zhengzhou University Zhengzhou 450001 China; g Department of Chemistry, University of Agriculture Faisalabad 38000 Pakistan rasheedahmadkhera@yahoo.com

## Abstract

Halide double perovskites have recently attracted attention as stable and environmentally benign alternatives to lead based perovskites for optoelectronic and energy applications. However, detailed insights into their stability, electronic structure, and multifunctional properties remain limited. In this study, the physical properties of Rb_2_AlInX_6_ (X = Cl, Br) were systematically examined by first-principles calculations. The structural stability of both compounds was confirmed through formation enthalpy, tolerance factor (*τ*_G_), octahedral factor (*µ*), and octahedral misfit (Δ*µ*), all of which fall within the accepted stability ranges. Both Rb_2_AlInCl_6_ and Rb_2_AlInBr_6_ crystallize in the cubic *Fm*3*m* phase with optimized lattice constants of 20.37 and 21.43 bohr, respectively. Electronic structure analysis identifies both Rb_2_AlInCl_6_ and Rb_2_AlInBr_6_ as semiconducting with calculated bandgaps of 2.85 eV and 1.90 eV, respectively, underscoring their potential for optoelectronic applications. Mechanical stability, verified *via* Born criteria, was further supported by elastic tensor analysis, demonstrating isotropic and robust mechanical behavior. The Rb_2_AlInBr_6_ exhibits moderate absorption extending into the visible region, while the Rb_2_AlInCl_6_ primarily absorbs in the near UV. These features suggest potential for optoelectronic or UV photodetection applications. While thermoelectric analysis shows notable power factor values at 800 K, pointing toward possible thermoelectric applications. These findings provide a comprehensive understanding of Rb_2_AlInX_6_ halides, offering valuable insights into their multifunctional prospects in next generation optoelectronic and thermoelectric devices.

## Abstract 2

First principle calculations reveal Rb_2_AlInCl_6_ and Rb_2_AlInBr_6_ as stable halide double perovskites with visible range bandgaps, strong UV-vis absorption, and promising thermoelectric (*ZT* > 0.7) and optoelectronic potential.

## Graphical abstract

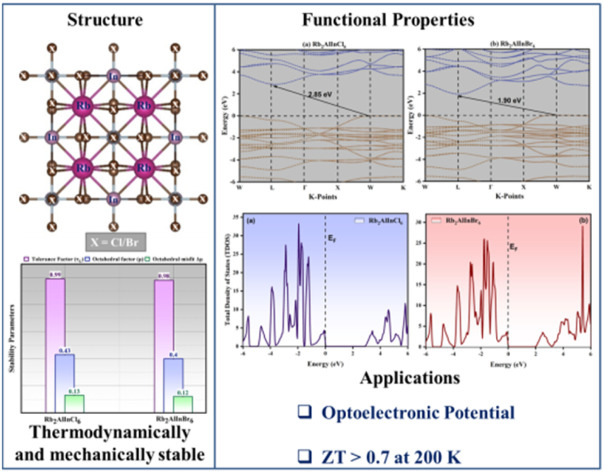

## Introduction

Scientists are finding novel materials that may be used to address the technical issues of the present time as technology advances, such as rechargeable battery packs that may be utilized at room temperature or above, and high stress bearing ceramics for the aviation industry.^1–3^ Owing to their remarkable potential and versatility, halide double perovskites are a major topic of interest for material scientists, who have made amazing strides in developing novel materials. The formula for HDPs is A_2_BB′X_6_, where X is a halogen ion, B and B′ represent ions from magnetic or other trivalent metals, and A stands for alkali or alkaline earth compounds (or related).^4,5^ Additionally, several studies have been provided to grasp the electronic, magnetic, and morphological features of HDPs. Rb_2_AgAlX_6_ (X = Br, I) demonstrated bandgap (*E*_g_) of 2.60 eV and 1.08 eV, which specifies that they may absorb light energy from the UV to VIS spectrum. Their potential for incorporation into cutting-edge solar energy systems is further highlighted by the absorption band of Rb_2_AgAsI_6_, from 1.7 eV to 3.4 eV.^6^ The direct *E*_g_ of K_2_InSbCl_6_ (1.31 eV) and K_2_InSbBr_6_ (1.22 eV) enables efficient absorption in the UV-vis regions, which makes these perfect for solar uses. Also, their excellent transport properties suggest strong potential for thermoelectric (TE) applications at 300 K.^7^ Additionally, it has been effectively forecast that many stable and ecologically friendly HDPs will be viable and sustainable substitutes for solar power and thermoelectric techniques, including Rb_2_YInX_6_ (X = Cl, Br, I),^8^ Cs_2_AgInX_6_ (X = F, Cl, Br, I),^9^ and Cs_2_InAgCl_6_.^10^ Cs_2_AgBi(Br, Cl)_6_ have indirect *E*_g_ of 2.19 and 2.77 eV, and show air stability with minimal degradation. These environmentally friendly semiconductors offer a suitable perovskite for sustainable devices.^11^ Several other related HDPs like Cs_2_ErXCl_6_ (X = Ag, Au).^12^ X_2_ScHgCl_6_ (X = Cs, Rb)^13^ and A_2_YHgCl_6_ (A = Cs, K)^14^ were also suggested as sustainable applications.

The lack of previous research on Rb_2_AlInX_6_ (X = Cl, Br) offers a chance to examine their physical characteristics. This study aims to provide valuable details into the fundamental properties of both compounds and their potential applications in advanced technologies through an in-depth analysis. Such pioneering work not only expands the knowledge base of halide double perovskites but also sets the stage for their integration into future technological innovations.

### Computational methods

DFT calculations were applied to investigate the electronic structure of the Rb_2_AlInX_6_ (X = Cl, Br).^15,16^ To guarantee accurate *E*_g_ analyses, the modified Becke–Johnson (mBJ) was used, as shown by the equation below^17^1
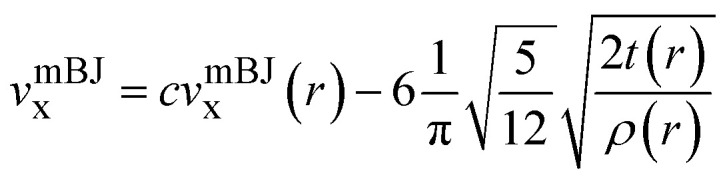


It employs the electron density to determine the electronic structure. These are solved using the FP-LAPW technique.^18^ The wave functions within each muffin-tin sphere are expanded using particular parameters in this technique. The *R*_MT_ and *K*_max_ products are set to 8 to guarantee accurate representation. A Monkhorst–Pack grid equivalent to 1000 *k*-points in the full Brillouin zone was employed to ensure accurate sampling. A convergence threshold of 0.00001 Ry for charge density was applied during self-consistent field (SCF) iterations, which ensured total energy convergence within 1 × 10^−5^ Ry. Energy-volume determinations were used to optimize the structure using the Murnaghan equation:^19^2
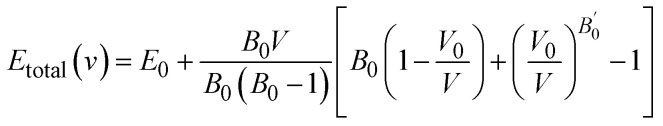


We examined the *E*_g_ dependent optical properties using the Kramers–Kronig relations. To calculate the muffin-tin radius (*R*_MT_), two conditions had to be met: (i) the MT spheres had to be free of core charge leakage, and (ii) there had to be no overlapping between the spheres. For Rb, Al, In, Cl, and Br, the *R*_MT_ values were 2.5, 2.15, 2.5, 2.1, and 2.38 bohr to ensure that there was no current loss. The elastic constants were computed separately using the CASTEP module in the Materials Studio package, employing a 4 × 4 × 4 Monkhorst–Pack *k*-point grid. Additionally, TE These properties were calculated using the BoltzTraP code,^20^ which employs the rigid band approximation (RBA) and the constant relaxation time approximation (CRTA). In the RBA, the effect of temperature and carrier concentration on the band structure is neglected, which may slightly affect the accuracy of Seebeck coefficient predictions at high temperatures.^21^ Similarly, the CRTA assumes a constant carrier scattering time (*τ*) independent of temperature and energy, whereas in halide perovskites, strong electron–phonon coupling can lead to *τ* variations that influence both conductivity and thermopower.^22–24^ These approximations, though widely used for qualitative trend analysis, may limit the precision of absolute transport coefficients. Although these approximations simplify the transport description, they remain reasonable and widely used for a first-order estimation of carrier transport.

## Results and discussion

### Structural features

**Fig. 1:**
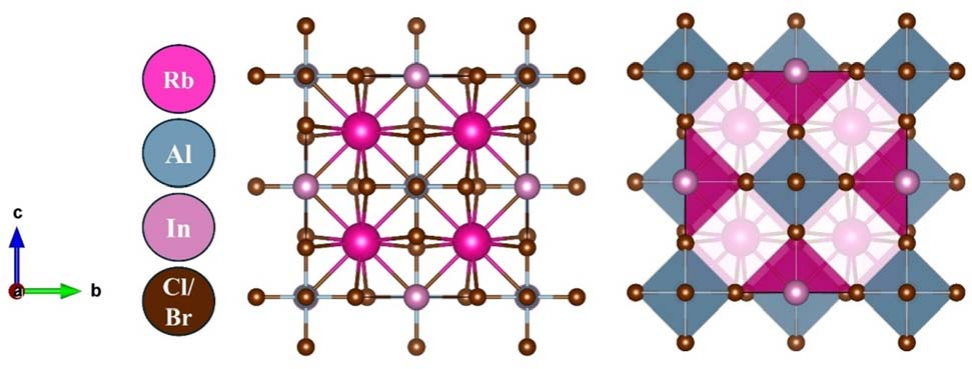
Crystal structure of Rb_2_AlInX_6_ (X = Cl, Br).

**Fig. 2:**
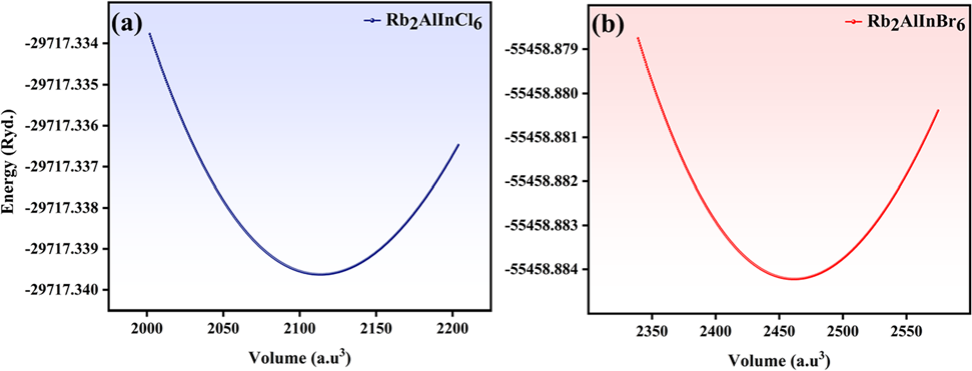
Optimization in non-magnetic phase of (a) Rb_2_AlInCl_6_ and (b) Rb_2_AlInBr_6_.

The structural analysis reveals that Rb_2_AlInX_6_ (X = Cl, Br) crystallizes in a cubic structure with space group *Fm*3*m* (No. 225). The structure of Rb_2_AlInX_6_ (X = Cl, Br) is illustrated in Fig. 1 with the atomic sites of Rb, Al, In, and X at (0.75,0.25,0.25), (0,0,0), (1/2,0,0), and (0.75,0,0), correspondingly. The enhanced lattice constants for Rb_2_AlInCl_6_ and Rb_2_AlInBr_6_ are determined to be 20.37 and 21.43 bohr, correspondingly. The structures optimization is illustrated in Fig. 2 and relaxation properties are displayed in Table 1 include lattice constants (Å), optimized bulk modulus (B), its derivative (Bp), ground state energy *E*_0_ (Ry), and volume.

**Table 1:** Calculated values of lattice constant (Å), *B* (GPa), Bp (GPa), volume (a.u),^3^ Δ*H* and ground state energies *E*_0_ (Ry) of stable state of cubic Rb_2_AlInX_6_ (X = Cl, Br)

Parameters	Rb_2_AlInCl_6_	Rb_2_AlInBr_6_	Rb_2_InSbCl_6_ (ref. 25)	Rb_2_InSbBr_6_ (ref. 25)
Lattice constant (Å)	10.780	11.342	11.23	11.78
*B* (GPa)	26.170	23.7137	23.11	19.69
Bp (GPa)	5.0	5.0	4.66	4.66
*V* (a.u.)^3^	2113.4876	2461.7013	—	—
*E*_0_ (Ry)	−29717.339	−55458.88	—	—
*τ*_G_	0.99	0.98	0.98	0.96
Δ*H* (eV)	−1.953	−3.247	−1.62	−1.34

A stable crystal structure is achieved by minimizing lattice strain and ensuring ideal ionic packing through a well-balanced tolerance factor. The distortion of the metal halide octahedra is also affected by octahedral misfit values, which can improve defect tolerance and adjust electronic band alignment. When constructing improved HDPs, these structural benefits are crucial since they greatly improve the material performance in optoelectronic applications.^26,27^ By applying the relation given below to calculate the formation enthalpy (Δ*H*), the thermodynamic integrity of both HDPs is verified.

3 Δ*H* = *E*_Rb_2_AlInX_6__ − 2*E*_Rb_ − *E*_Al_ − *E*_In_ − 6*E*_X_

Δ*H* needs to be negative to be thermodynamically stable. The calculated Δ*H* values for Rb_2_AlInCl_6_ are −1.953 and −3.247 eV for Rb_2_AlInBr_6_, signifying that both are robust and unlikely to degrade under typical conditions. Recent investigations on Cs_2_InAsX_6_ (X = Cl, Br) have shown that both are stable and exhibit negative Δ*H* values of −2.20 and −3.64 eV.^28^ Furthermore, X_2_LiSbI_6_ (X = K, Cs) compounds have been reported to be dynamically stable, exhibiting negative formation energies of −3.88 eV and −3.95 eV, respectively.^29^

The stability factors like *τ*_G_, *µ*, and Δ*µ* are calculated to determine the stability of Rb_2_AlInCl_6_ and Rb_2_AlInBr_6_.^26,27^ These are determined as:4
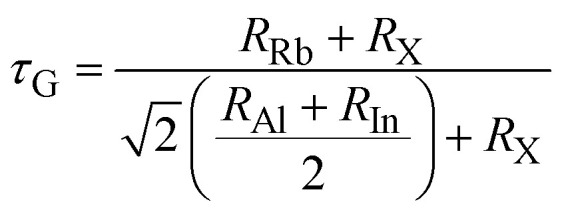
5
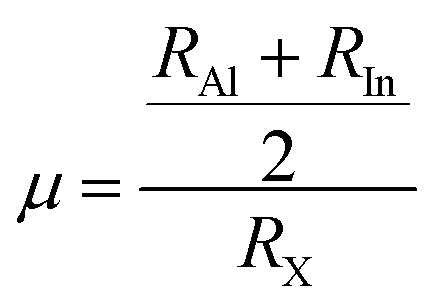
6
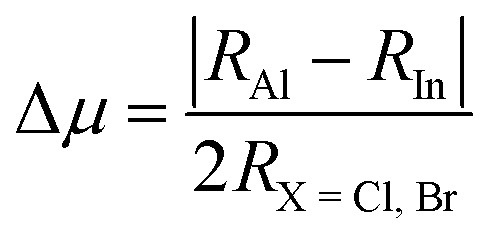


**Fig. 3:**
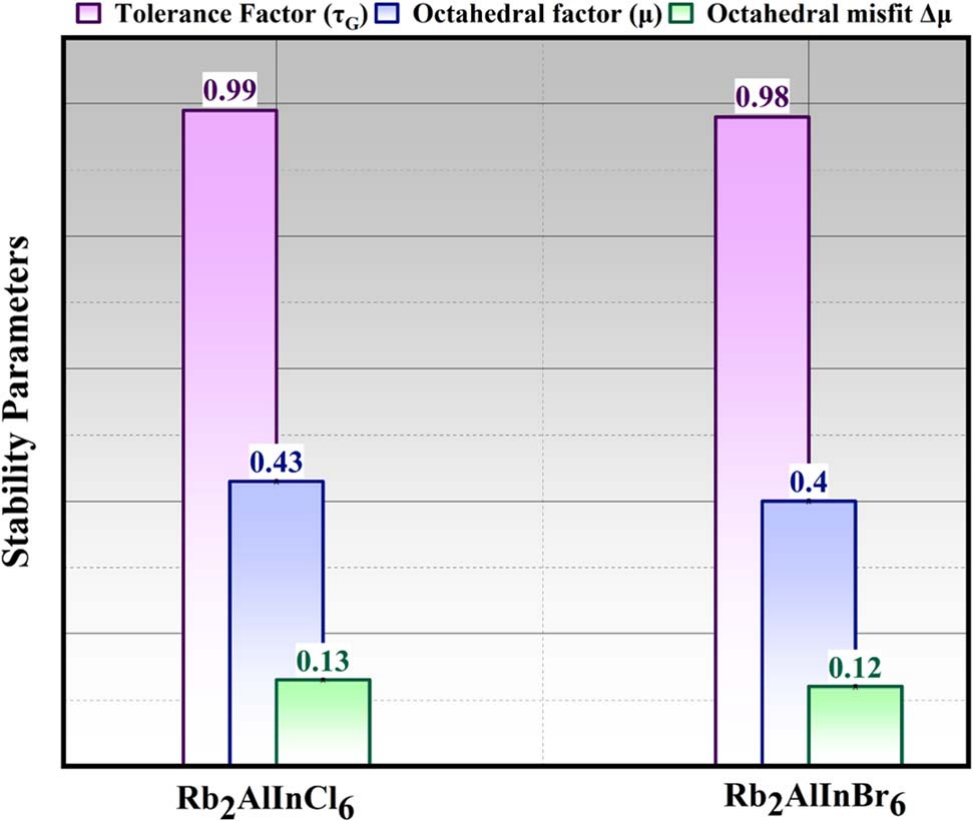
Graphical representation of stability Parameters of Rb_2_AlInX_6_ (X = Cl, Br).

These are determined using ionic radii values of Rb, Al, In, and Cl/Br shown as *R*_Rb_, *R*_Al_, and the mean of *R*_Cl_ and *R*_Br_. *τ*_G_ range for stable perovskites is 0.8 to 1, with unity values indicating perfect structure. The determined values of this factor are 0.99 and 0.98 for Rb_2_AlInCl_6_ and Rb_2_AlInBr_6_, confirming their stability. To confirm the validity of these results, a comparison with analogous compounds Cs_2_XCeI_6_ (X = Li, Na) from Murtaza *et al.*^30^ showed *τ* values of 0.87 and 0.85 for Cs_2_LiCeI_6_ and Cs_2_NaCeI_6_. The successful synthesis of several related perovskites including Cs_2_InBiCl_6_, Cs_2_InBiBr_6_, Cs_2_InBiI_6_,^31^ Cs_2_ScAgI_6_,^32^ Cs_2_NaLaCl_6_,^33^ Cs_2_YAuBr_6_,^34^ K_2_InBiBr_6_,^35^ and Cs_2_LiCeF_6_ (ref. 36) further supports their structural stability in agreement with the Goldschmidt tolerance factor model. The Δ*µ* values are computed as 0.13 and 0.12 for Cl and Br perovskites, showing their stability as both are closer to the null value.^37^ Moreover, the stability of these perovskites is also validated from *µ* values, which are calculated as 0.43 and 0.40 and exist within the standard stability range of 0.4 to 0.9 as shown in Fig. 3.^37,38^

### Electronic properties

**Fig. 4:**
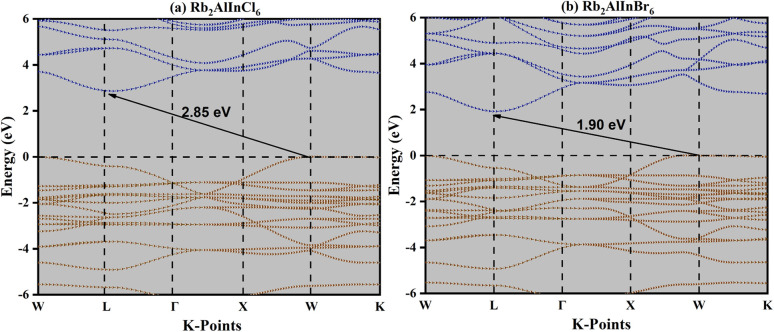
Band structure of (a) Rb_2_AlInCl_6_ and (b) Rb_2_AlInBr_6_.

To comprehend the electronic and optical characteristics Rb_2_AlInCl_6_ and Rb_2_AlInBr_6_, the electronic band structures (BS) have been examined in conjunction with their structural characteristics. By combining information about crystal structure and electronic properties, BS provides information about the conduction and basic electronic nature of halides.^39^ mBJ potentials were used to calculate the BS for Rb_2_AlInCl_6_ and Rb_2_AlInBr_6_. The indirect *E*_g_ values of 2.85 eV for Rb_2_AlInCl_6_ and 1.90 eV for Rb_2_AlInBr_6_ are calculated. As the atomic radius increases from Cl to Br, this trend shows a drop in *E*_g_, as shown in Fig. 4. To further check the relativistic effects, spin orbit coupling (SOC) was incorporated into the mBJ calculations. The inclusion of SOC slightly reduced the *E*_g_ values to 1.82 eV for Rb_2_AlInCl_6_ and 1.18 eV for *E*_g_, without altering the indirect nature of the *E*_g_. The overall band dispersion and density of states (DOS) features remained qualitatively similar, confirming that SOC has only a minor influence on the electronic structure of these compounds. The corresponding SOC band structure and DOS plots are provided in the SI (Fig. S1). To assess the accuracy of our *E*_g_ calculations, we compared them with experimental *E*_g_ of well-known double perovskites. For instance, Cs_2_BiAgCl_6_ displayed a *E*_g_ of 2.2 eV, while Cs_2_AgBiBr_6_ showed a *E*_g_ of 1.95 eV.^40,41^ Our predicted values are in agreement with experimental data, with small discrepancies. This comparison confirms the reliability of our *E*_g_ predictions, providing quantitative error bars for the *E*_g_. These values are consistent with those observed in the literature and remain applicable for further exploration of related thermoelectric properties.

The total density of state (TDOS) and partial density of states (PDOS) for Rb_2_AlInCl_6_ and Rb_2_AlInBr_6_ reveal important insights into their electronic structures. In the valence band (VB) region, the In-5p orbitals show a notable contribution, indicating their strong bonding interactions with the halide atoms. For Rb_2_AlInBr_6_, the Br-4p orbitals dominate the VB and display strong hybridization with the In-5p orbitals, highlighting robust bonding. Similarly, in Rb_2_AlInCl_6_, the Cl-3p orbitals show a critical role in the VB, with prominent hybridization peaks aligning with the In-5p orbitals. Rb-5s states show negligible contribution, consistent with their non-bonding nature. Al-3p states contribute weakly near the Fermi level, indicating a minor influence on the electronic structure. In the conduction band, contributions from the In-5p orbitals dominate, along with significant involvement from Br4p in Rb_2_AlInBr_6_ and Cl-3p in Rb_2_AlInCl_6_, indicating their crucial role in optical excitation and conductivity.

**Fig. 5:**
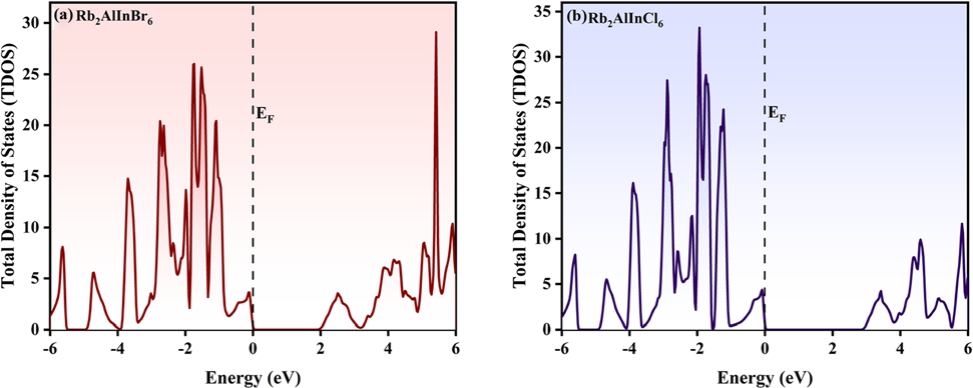
Density of states (TDOS) profile of (a) Rb_2_AlInCl_6_ and (b) Rb_2_AlInBr_6_.

**Fig. 6:**
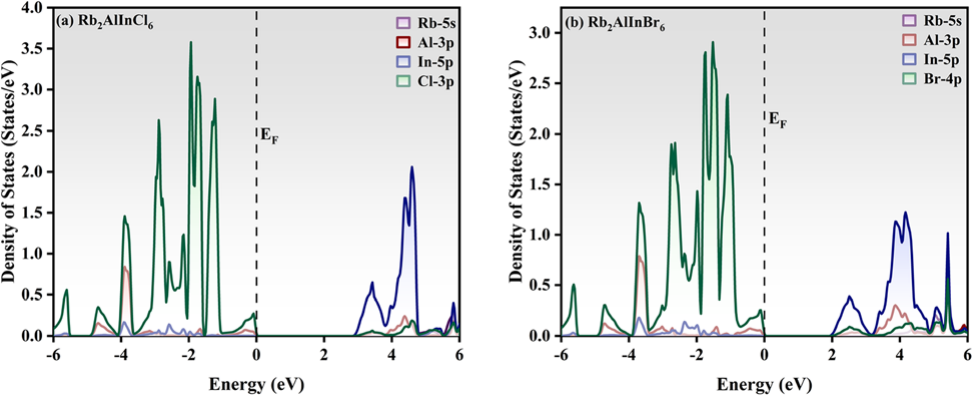
Partial density of states (PDOS) profile of (a) Rb_2_AlInCl_6_ and (b) Rb_2_AlInBr_6_.

The TDOS and PDOS profile of Rb_2_AlInCl_6_ and Rb_2_AlInBr_6_ is shown in Fig. 5 and 6. The changing of Br with Cl leads to a narrower valence band is observed for Rb_2_AlInCl_6_ due to the smaller ionic radius of chlorine, which strengthens bonding and pushes the valence states to slightly deeper energies, whereas the larger ionic radius of bromine in Rb_2_AlInBr_6_ leads to a broader valence band with enhanced hybridization effects. Both compounds exhibit semiconducting behaviour, with the CB dominated by In-5p and halide p-orbitals, underlining their potential for optoelectronic uses.

### Optical properties

**Fig. 7:**
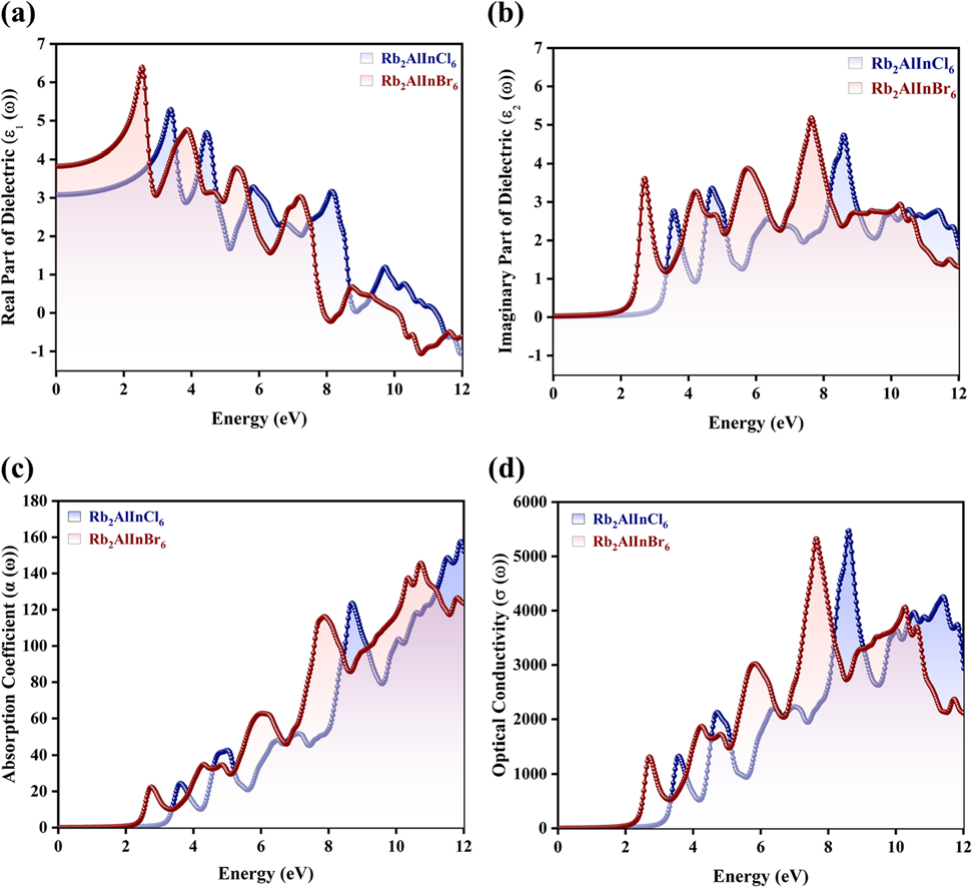
Optical parameters (a) real *ε*_1_(*ω*) component, (b) imaginary *ε*_2_(*ω*) component, (c) absorption coefficient *α*(*ω*) and (d) optical conductivity *σ*(*ω*) for Rb_2_AlInX_6_ (X = Cl, Br).

A primary consideration in determining whether solar cells are feasible for energy generation is their efficiency. The material optical characteristics, which control how it interacts with incoming electromagnetic (EM) radiation, are directly related to it.^42,43^ The dielectric function *ε*(*ω*) is an intricate function that controls the relationship between a photovoltaic material and incoming EM light. It is important in this context.^44–46^ The real *ε*_1_(*ω*) and imaginary *ε*_2_(*ω*) components of the *ε*(*ω*) can be used to obtain additional optical characteristics that further influence the material's solar power capacity. These characteristics include the absorption coefficient *α*(*ω*), refractive index *n*(*ω*), reflectivity *R*(*ω*), extinction coefficient *k*(*ω*), optical conductivity *σ*(*ω*), and energy loss function *L*(*ω*). Scattering information is provided by *ε*_1_(*ω*), while absorption attributes are provided by *ε*_2_(*ω*).^47,48^ The *ε*_1_(*ω*) describes the degree of photon scattering and the transmission speed, which is dependent on the largest light dispersion. The static *ε*_1_(0) values for Rb_2_AlInCl_6_ and Rb_2_AlInBr_6_ are 3.15 and 3.91, respectively (Fig. 7(a)). It's interesting to note that this validation of Penn's model shows that *E*_g_ and *ε*_1_(0) have the opposite connection^49^ as shown in eqn (7):7
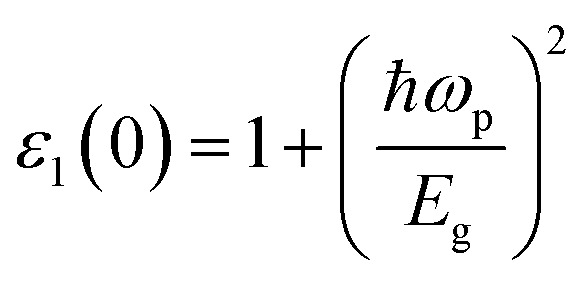


A higher *ε*_1_(0) indicates a stronger interaction with the electromagnetic field and enhanced polarization under an external electric field, suggesting improved photon–electron coupling efficiency. For photodetectors and solar cells uses, a larger *ε*_1_(0) value is associated with increased dielectric screening and better charge separation.^50,51^ Therefore, the relatively higher *ε*_1_(0) of Rb_2_AlInBr_6_ implies stronger light–matter interaction in the visible region, while the smaller *ε*_1_(0) of Rb_2_AlInCl_6_ points to faster photon transmission and suitability for UV and high frequency optoelectronic applications.

Before displaying peaks, the *ε*_2_(*ω*) spectra for Rb_2_AlInCl_6_ and Rb_2_AlInBr_6_ first show a fluctuating pattern with first peaks at 3.55 eV and 2.70 eV. Some researchers calculated static *ε*_1_(0) for the similar compounds. *ε*_1_(0) values of 7.00, 3.80, 2.5, and 3.2, respectively were calculated for Cs_2_ScInBr_6_, Cs_2_ScCuCl_6_, and Cs_2_ScCuF_6_,^52^ Cs_2_AuInCl_6_,^53^ whereas Cs_2_LiMoX_6_ (X = Cl, I)^54^ are 4.84 and 3.2037. Na_2_AuInCl_6_, Na_2_AuInBr_6_, and Na_2_AuInI_6_, it is calculated as 2.15, 2.34, and 3.75, respectively.^55^ The *ε*_2_(*ω*) component represents the optical absorption process, revealing how efficiently the material can absorb incident photons and promote interband electronic transitions. The *ε*_2_(*ω*) exhibits peaks at 8.63 eV for Rb_2_AlInCl_6_ and 7.64 eV for Rb_2_AlInBr_6_, producing identical results (Fig. 7(b)). The *ε*_1_(*ω*) trend across related perovskites indicates that Cs_2_InSbCl_6_ attains the highest value at 0.88 eV, with Cs_2_InSbBr_6_ and Cs_2_InSbI_6_ exhibiting increasing responses at 1.43 eV (5.9) and 2.87 eV (8.7), respectively, reflecting the influence of halide substitution on optical behavior.^56^ The sharp peaks in *ε*_2_(*ω*) confirm direct allowed transitions in both compounds, essential for visible and UV optoelectronics. However, the lower transition energy (2.70 eV) in Rb_2_AlInBr_6_ falls directly within the visible-light region, confirming its potential as a photoactive and emissive material for visible light photodetectors and LEDs.^57,58^ In contrast, Rb_2_AlInCl_6_ shows its main transitions at higher energies (3.55 eV and above), suggesting it could serve effectively in UV photodetectors, transparent window layers, or protective coatings in optoelectronic devices.^59^ The following equation can be used to compute it:^60^8
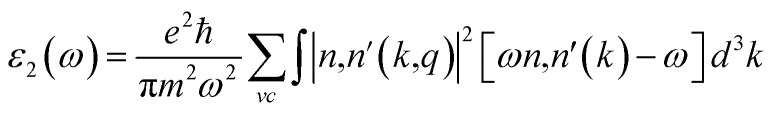


The high *α*(*ω*) in the visible and near UV regions^61^ demonstrates strong optical activity and efficient photon utilization.^62^ Such high absorption is a key requirement for photoactive absorber layers in solar cells and photodetectors, as it enables efficient electron–hole generation even in thin films.^63,64^ Both Rb_2_AlInCl_6_ and Rb_2_AlInBr_6_ show considerable values throughout a wide energy range when the *α*(*ω*). Both Rb_2_AlInCl_6_ and Rb_2_AlInBr_6_ exhibit significant optical transitions at 8.68 eV and 7.90 eV, respectively, in Fig. 7(c) corresponding to the deep UV region. In addition, the Rb_2_AlInBr_6_ compound shows additional weaker absorption features at lower energies, indicating a modest extension of optical activity into the visible region. This suggests that while both systems are primarily UV absorbers, the Rb_2_AlInBr_6_ possesses relatively enhanced visible light response, making it more promising for optoelectronic or photocatalytic applications. The movement of photoelectrically produced photons inside the substance is analysed by the *σ*(*ω*) and calculated as:9
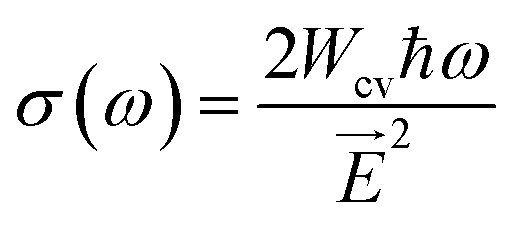


**Fig. 8:**
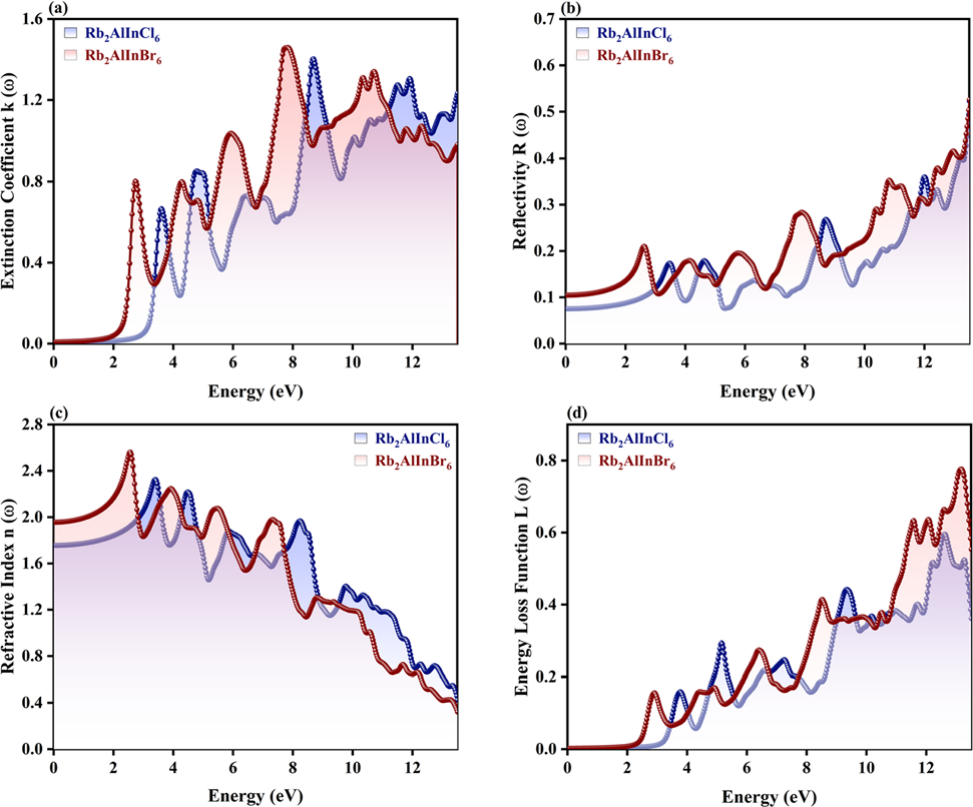
Optical features of Rb_2_AlInX_6_ (X = Cl, Br). (a) Extinction coefficient *k*(*ω*), (b) reflectivity *R*(*ω*), (c) refractive index *n*(*ω*) and (d) energy loss function *L*(*ω*).

The disruption of connections can be explained by the presence of strong EM radiation. *σ*(*ω*) exhibits free carriers produced upon capturing electromagnetic radiation, and that *σ*(*ω*) and *α*(*ω*) are intimately associated. A maximum *σ*(*ω*) reflects efficient carrier transport and reduced recombination losses, both vital for high performance optoelectronic devices.^65,66^ The *σ*(*ω*) peaks for Rb_2_AlInBr_6_ and Rb_2_AlInCl_6_ reach 5286 and 5534 Ω^−1^ cm^−1^ at 7.64 eV and 8.59 eV, respectively, indicating strong light induced carrier generation as shown in Fig. 7(d). The superior *σ*(*ω*) in Rb_2_AlInBr_6_ at lower photon energies suggests its carriers can be effectively excited under visible illumination, while Rb_2_AlInCl_6_, responding mainly to higher energy photons, is suitable for UV or high energy optoelectronics.^67^ Together, their strong *σ*(*ω*) and *α*(*ω*) responses confirm that both compounds can function as efficient charge transport and photon conversion layers in multi spectral optoelectronic systems.^68^ While our DFT-based calculations provide a reliable estimate of the fundamental *E*_g_, they do not account for excitonic effects, which are known to be significant in double perovskites with large exciton binding energies on the order of a few hundred m eV.^69^ These effects may result in a lower optical gap compared to the DFT calculated fundamental gap.^70^ However, our calculations still provide valuable insight into the material's electronic structure, and the predicted *E*_g_ remain applicable for general analysis of the material's properties. The combination of suitable *E*_g_, high absorption, and strong conductivity indicates that Rb_2_AlInBr_6_ is more efficient for visible light optoelectronic devices such as solar absorbers, LEDs, and photodetectors, while Rb_2_AlInCl_6_, with its wider gap, can play a complementary role in UV optoelectronics and as a transparent or electron blocking layer in heterojunction structures. Further investigations incorporating excitonic effects, such as many body perturbation theory (*e.g.*, GW approximation), would enhance the accuracy of optical absorption predictions.^71,72^*k*(*ω*) is another optical characteristic that is strongly associated with *α*(*ω*). The degree of damping of input photons *k*(0) in the alloys studied is *k*(*ω*), which is brought on by both dispersion and captivation. Importantly, because they are connected by Kramers–Kronig relations, it is similar to the *ε*_2_(*ω*).^73,74^ The most significant values for Rb_2_AlInCl_6_and Rb_2_AlInBr_6_ emerge at 8.70 eV and 7.76 eV in the fluctuating pattern of the *k*(*ω*) spectrum (Fig. 8(a)). The overall lower extinction coefficient of Rb_2_AlInBr_6_ in the visible range minimizes optical damping and photon loss, which is beneficial for light emitting and absorbing devices, whereas the higher *k*(*ω*) in Rb_2_AlInCl_6_ contributes to its efficiency in UV photon absorption and filtering. A key indicator of the proportion of EM radiation reflecting at a particular energy is the *R*(*ω*), which is displayed in Fig. 8(b).^75,76^ The *R*(*ω*) values were notable, first and foremost; for *ω* = 0, they were 0.08 and 0.10 for Rb_2_AlInCl_6_ and Rb_2_AlInBr_6_. As energy rises, *R*(*ω*) exhibits a fluctuating trend. *R*(*ω*) is calculated using the provided relation, and the greatest reflectance for Rb_2_AlInCl_6_ is shown at 8.68 eV and 7.81 eV for Rb_2_AlInBr_6_. *R*(*ω*) can be computed using the relation given below:10
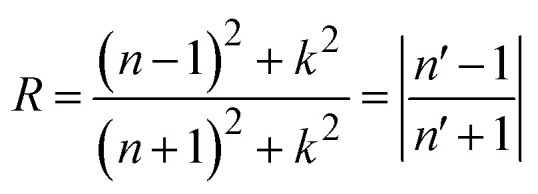


Moderate reflection at low energies and increasing reflection at higher photon energies indicate balanced optical behavior for both compounds. Rb_2_AlInBr_6_, with higher reflectivity in the visible region, is promising for light emitting and laser applications, while Rb_2_AlInCl_6_, exhibiting lower reflection and higher transmission, can be applied in anti-reflective or UV transparent coatings.^77^ The *n*(*ω*) varies as does *ε*_1_(*ω*), an important factor for evaluating substance transparency.^78^ As for Rb_2_AlInCl_6_ and Rb_2_AlInBr_6_, their respective static *n*(0) values are 1.78 and 1.97. According to Fig. 8(c), there are other noteworthy *n*(*ω*) peaks for Rb_2_AlInCl_6_ at 3.34 eV and for Rb_2_AlInBr_6_ at 2.53 eV. The HDP analysis revealed nearly identical results. The *n*(0) values of Cs_2_NaMoCl_6_ and Rb_2_NaMoCl_6_ were found to be 1.71 and 1.69,^79^ while Rb_2_YAuI_6_ and Cs_2_YAuI_6_ demonstrated slightly higher values of 2.01 and 2.03, consistent with their heavier halide composition.^80,81^ The slightly higher refractive index of Rb_2_AlInBr_6_ in the visible range ensures stronger photon confinement and efficient light–matter interaction, ideal for LEDs and photovoltaic absorbers.^82,83^ Conversely, the lower *n*(*ω*) of Rb_2_AlInCl_6_ improves transparency and is suitable for UV photonics or as a top layer coating in tandem solar architectures.^84^ The *L*(*ω*) shows the drop in photon energy as it passes through the material. The *L*(*ω*) peaks for Rb_2_AlInCl_6_ and Rb_2_AlInBr_6_ are seen at 9.32 eV and 8.56 eV (Fig. 8(d)). A greater *L*(*ω*) value corresponds to more pronounced plasmonic resonance and stronger collective electron oscillations. The lower energy loss of Rb_2_AlInBr_6_ indicates less internal damping and greater photon utilization in the visible region, making it favourable for LEDs and photodetectors.^85^ Meanwhile, Rb_2_AlInCl_6_, with a higher *L*(*ω*) and wider *E*_g_, is suitable for UV sensing and protective optical devices. Therefore, it can be concluded that Rb_2_AlInBr_6_, with its higher absorption coefficient, greater optical conductivity, and favourable refractive behaviour, is the most promising compound for visible light optoelectronic applications, while Rb_2_AlInCl_6_ can serve complementary roles in UV detection, high frequency photonics, and as a transparent coating layer.^86,87^

### Thermoelectric features

**Fig. 9:**
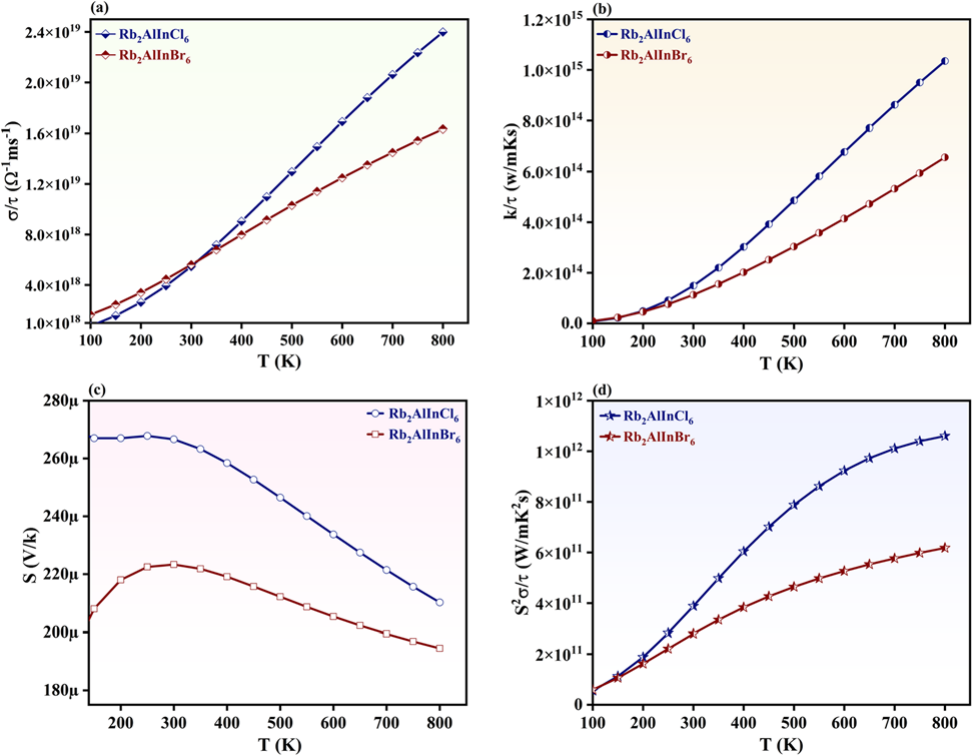
Thermoelectric factors (a) electrical conductivity (*σ*/τ), (b) thermal conduction (*k*/*τ*) (c) Seebeck coefficient (*S*) and (d) power factor of Rb_2_AlInX_6_ (X = Cl, Br).

HDPs exhibit promising TE properties because of their exceptional electronic structures, favourable carrier transport mechanisms, and tunable *E*_g_. Their TE performance is primarily governed by a delicate balance between electrical conductivity (*σ*/*τ*), Seebeck coefficient (*S*), and thermal conductivity (*k*/*τ*).^88,89^ Using the BoltzTraP algorithm,^20^ the efficacy of Rb_2_AlInCl_6_ and Rb_2_AlInBr_6_ as TE materials is assessed. Fig. 9 presents the temperature dependent factors governing the thermoelectric performance of Rb_2_AlInCl_6_ and Rb_2_AlInBr_6_. The electronic components of *k*/*τ* are identified by the BoltzTraP code by removing the contributions from holes. A predetermined relaxation period (*τ*) of 10^−14^ seconds for TE characteristics is assumed in the computations. Results demonstrate a significant power factor (PF), low *k*/*τ*, and high *σ*/*τ*, which suggest strong TE efficiency. The quantity and motion of carriers are influenced by the *σ*/*τ*. Since higher *T* offers them the energy they need to shift, conductivity and temperature are directly correlated.^42,90^ The *σ*/*τ* increases nearly linearly with temperature for both compounds, with Rb_2_AlInCl_6_ consistently higher than Rb_2_AlInBr_6_ across the entire range. At 800 K, *σ*/*τ* reaches 2.3 × 10^19^ Ω^−1^ m s^−1^ for Rb_2_AlInCl_6_ and 1.5 × 10^19^ Ω^−1^ m s^−1^ Rb_2_AlInBr_6_ as depicted in Fig. 9(a). In comparison to similar halide perovskites, this series of compounds demonstrates competitive room-temperature electric conductivity, with recorded values of 0.29 × 10^18^, 0.21 × 10^18^, 0.25 × 10^18^, and 0.24 × 10^18^ Ω^−1^ m s^−1^ for K_2_TlBiCl_6_, K_2_TlBiBr_6_, Rb_2_TlBiCl_6_, and Rb_2_TlBiBr_6,_ respectively.^91^

The *k*/*τ* value quantifies a material's ability to conduct heat.^92,93^*k*/*τ* grows constantly with temperature. The *k*/*τ* values increase with temperature, with Rb_2_AlInCl_6_ showing higher values than Rb_2_AlInBr_6_. At 800 K, *k*/*τ* reaches 1.05 × 10^15^ W m^−1^ K^−1^ s^−1^ for Rb_2_AlInCl_6_ and 7.5 × 10^14^ W m^−1^ K^−1^ s^−1^ Rb_2_AlInBr_6_ as depicted in Fig. 9(b). Compared to similar materials, K_2_AlInF_6_, K_2_AlInCl_6_, and K_2_AlInBr_6_, when measured at 800 K, not only exhibit Seebeck coefficients of 150, 160, and 135 µV K^−1^, respectively, but also a significant rise in *k*/*τ* to between 4.0–4.75 × 10^14^ W m^−1^ K^−1^ s^−1^.^94^

*S* plays a key role in determining thermoelectric efficiency.^95–97^ The primary carriers are holes, as shown by a positive reaction in the *S* spectrum.^98^ This verifies the p-type nature of Rb_2_AlInCl_6_ and Rb_2_AlInBr_6_. For Rb_2_AlInCl_6_ and Rb_2_AlInBr_6_, *S* decreases across the whole *S* spectrum (Fig. 9(c)). For Rb_2_AlInCl_6_, the maximum value of *S* is 209 µV K^−1^ at 200 K, suggesting the TE potential of both HDPs in various uses at lower temperatures. The PF has a major effect on TE performance and can be computed as PF = *S*^2^*σ*/*τ*.^99^ For Rb_2_AlInCl_6_ and Rb_2_AlInBr_6_, the lowest PF at 200 K is 1.35 × 10^11^ W K^−2^ m^−1^ s^−1^. As the *T* rises, both HDP materials show a steadily growing trend in the PF plot (Fig. 9(d)). The most notable PF values for Rb_2_AlInCl_6_ and Rb_2_AlInBr_6_ at 800 K are 1.0 × 10^12^ W K^−2^ m^−1^ s^−1^ and 6.01 × 10^11^ W K^−2^ m^−1^ s^−1^. Thermoelectric analysis of related perovskites shows that Cs_2_ScTiCl_6_ achieves a PF of 2.6 × 10^−2^ W m^−1^ K^−2^ s^−1^ at 800 K, closely followed by Cs_2_YTiCl_6_ with 2.3 × 10^−2^ W m^−1^ K^−2^ s^−1^.^100^ The higher PF values obtained for Rb_2_InSbCl_6_ (4.5 × 10^10^ W m^−1^ K^−2^ s^−1^) and Rb_2_InSbBr_6_ (2.5 × 10^10^ W m^−1^ K^−2^ s^−1^) further confirm their potential as promising thermoelectric materials.^100^ The outstanding TE characteristics of Rb_2_AlInCl_6_ and Rb_2_AlInBr_6_ suggest that these compounds would be enormous options for TE generators and cooler devices.

## Conclusion

In this study, the structural, electronic, optical, mechanical, and thermoelectric properties of Rb_2_AlInX_6_ (X = Cl, Br) halide double perovskites were comprehensively investigated using density functional theory. The electronic and optical properties were computed using the WIEN2k code with the mBJ potential for accurate estimation of *E*_g_, while the elastic constants were calculated using the CASTEP module to validate mechanical stability. Both compounds crystallize in the stable cubic *Fm*3*m* phase and exhibit indirect semiconducting *E*_g_ of 2.85 eV for Rb_2_AlInCl_6_ and 1.90 eV for Rb_2_AlInBr_6_. TDOS and PDOS analyses reveal strong hybridization between In-5p and halogen-p orbitals, confirming a mixed covalent-ionic bonding nature responsible for their semiconducting behaviour. The calculated elastic constants satisfy the Born mechanical stability criteria and indicate ductile mechanical behaviour, suggesting high structural integrity under external stress. Optical analysis reveals pronounced absorption in the UV-visible region, strong dielectric response, and high optical conductivity, indicating efficient light–matter interaction suitable for optoelectronic applications. Thermoelectric calculations performed using the BoltzTraP code reveal enhanced power factors and moderate thermal conductivities, resulting in *ZT* values of 0.78 for Rb_2_AlInCl_6_ and 0.70 for Rb_2_AlInBr_6_ at 200 K. Collectively, the results demonstrate that Rb_2_AlInCl_6_ and Rb_2_AlInBr_6_ are mechanically and thermodynamically stable lead-free halide double perovskites, exhibiting excellent prospects for integration into high performance optoelectronic and thermoelectric energy harvesting devices.

## Author contributions

S. M. K. A. Naqvi and R. A. Khera conceived and designed the study; S. Abdalla and K. Akhtar carried out the computational modelling and figure preparation; A. Ali and N. Y. Elamin contributed to data analysis and interpretation; S. M. K. A. Naqvi, F. Abbas and A. M. Khan participated in writing and editing the manuscript.

## Conflicts of interest

The authors declare that they have no known competing financial interests or personal relationships that could have appeared to influence the work reported in this paper.

## Supplementary Material

RA-015-D5RA06712J-s001

RA-015-D5RA06712J-s002

RA-015-D5RA06712J-s003

RA-015-D5RA06712J-s004

RA-015-D5RA06712J-s005

RA-015-D5RA06712J-s006

RA-015-D5RA06712J-s007

## Data Availability

All data provided and/or analysed during this study were included as figures and tables in this article. Supplementary Information: additional results, including detailed mechanical properties and band structure/DOS graphs with spin–orbit coupling (SOC) effects. See DOI: https://doi.org/10.1039/d5ra06712j.
