# Supplementary figures and images for: Exploring lead free Rb2AlInX6 halide double perovskites for advanced energy harvesting applications
Source: RSC Adv. 2025 Nov 12;15(52):44116–24. doi: 10.1039/d5ra06712j (PMC12608080; doi:10.1039/d5ra06712j)

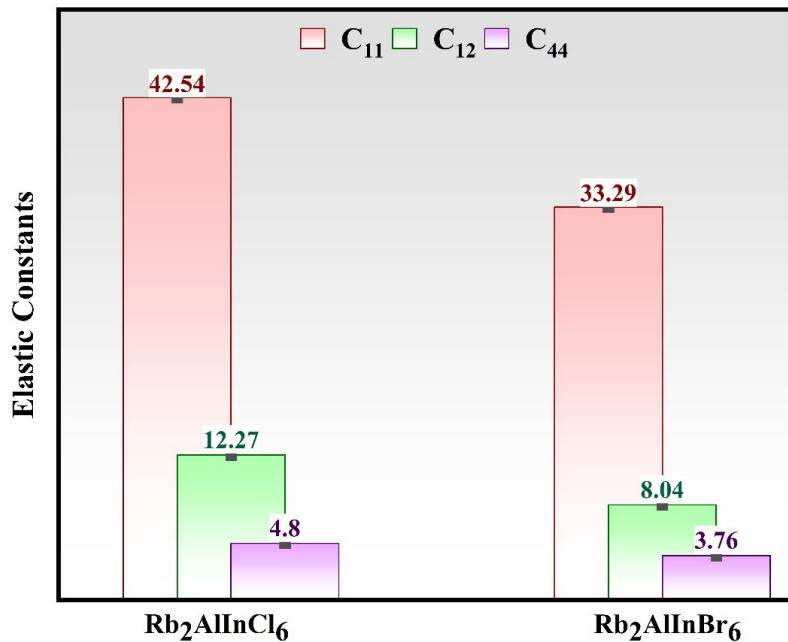

Supplement: RA-015-D5RA06712J-s002 [file RA-015-D5RA06712J-s002.pdf]

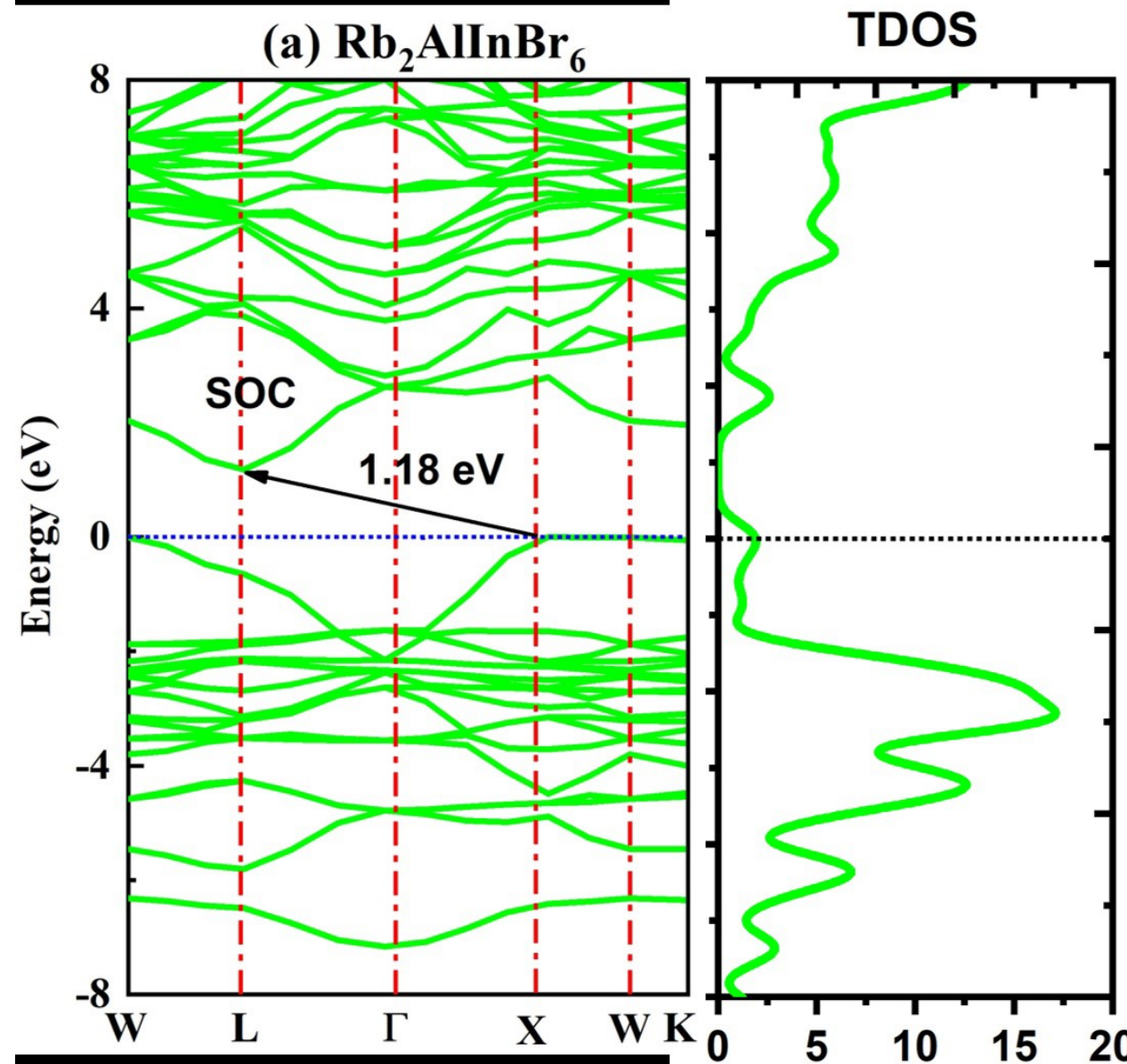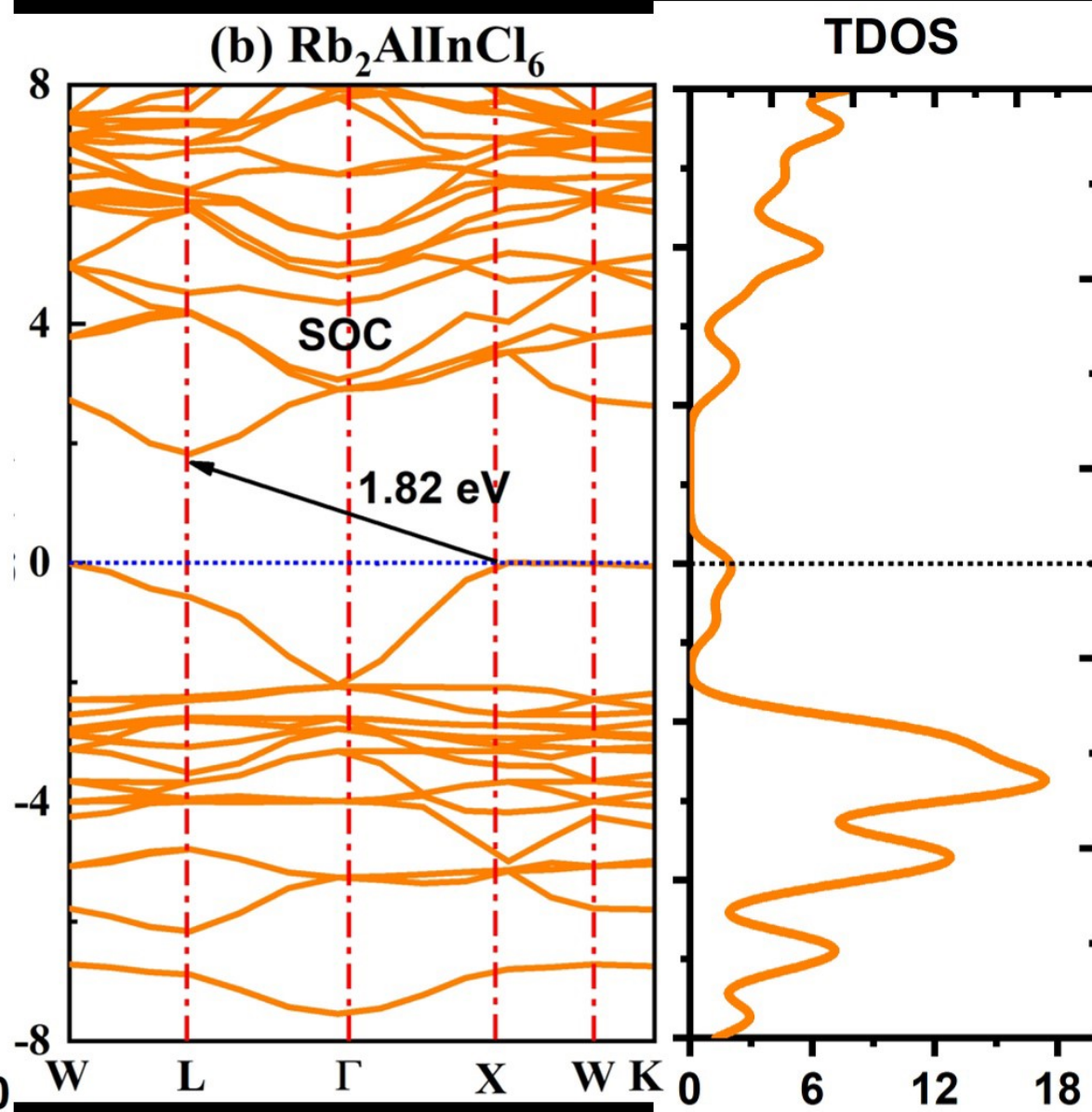

Supplement: RA-015-D5RA06712J-s005 [file RA-015-D5RA06712J-s005.pdf]
