# Supplementary material for: Exploring lead free Rb2AlInX6 halide double perovskites for advanced energy harvesting applications
Source: RSC Adv. 2025 Nov 12;15(52):44116–24. doi: 10.1039/d5ra06712j (PMC12608080; doi:10.1039/d5ra06712j)

Supplementary Information (SI) for RSC Advances.
This journal is © The Royal Society of Chemistry 2025

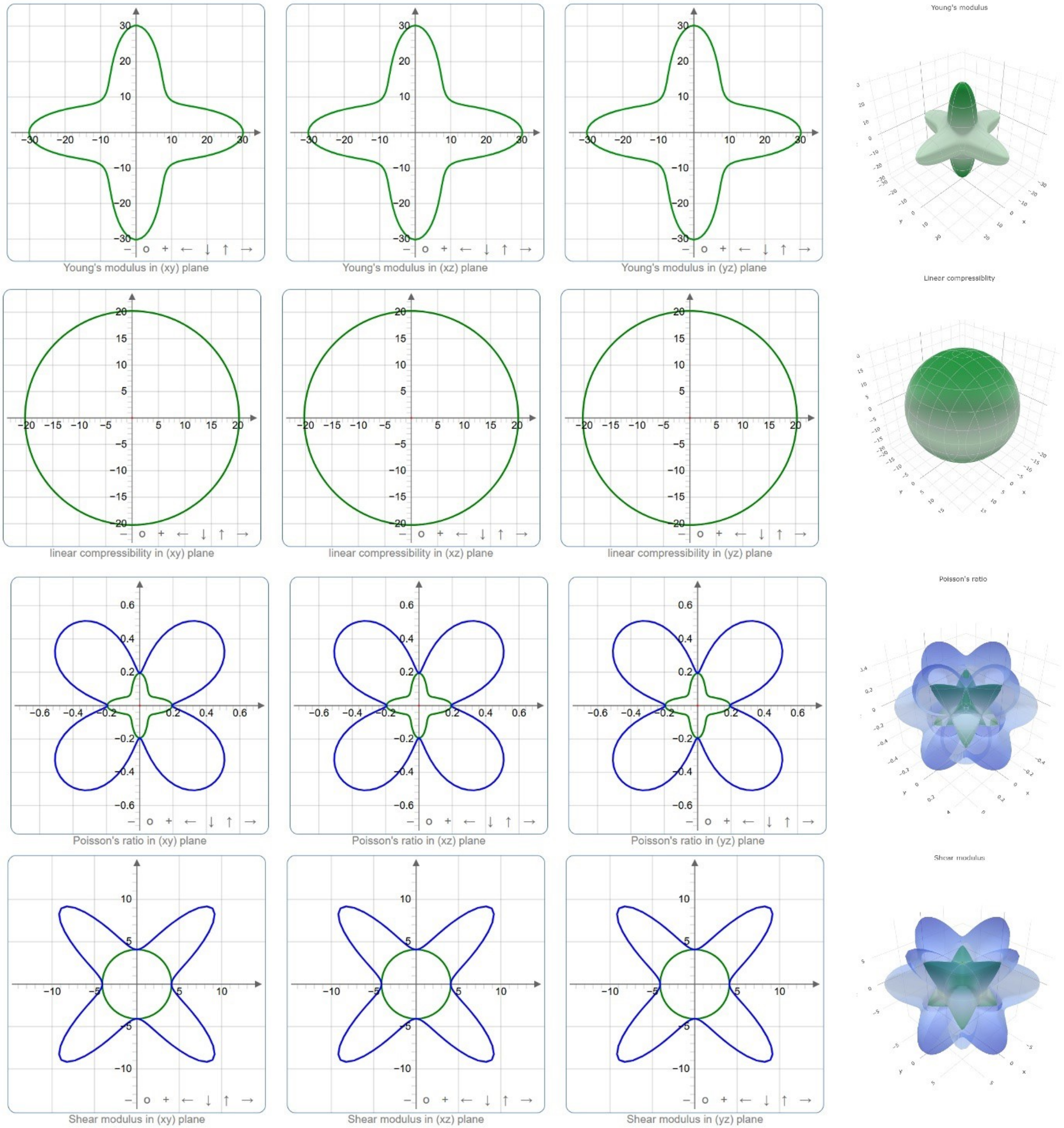

Supplement: RA-015-D5RA06712J-s004 [file RA-015-D5RA06712J-s004.pdf]
