# Supplementary material for: Exploring lead free Rb2AlInX6 halide double perovskites for advanced energy harvesting applications
Source: RSC Adv. 2025 Nov 12;15(52):44116–24. doi: 10.1039/d5ra06712j (PMC12608080; doi:10.1039/d5ra06712j)
Supplement: RA-015-D5RA06712J-s007 [file RA-015-D5RA06712J-s007.pdf]

Supplementary Information (SI) for RSC Advances.
This journal is © The Royal Society of Chemistry 2025

Exploring Lead Free Rb_2AlInX_6 Halide Double Perovskites for Advanced Energy Harvesting Applications

Syed Muhammad Kazim Abbas Naqvi,[a] Sahar Abdalla,[b] Kiran Akhtar,[c] Aleesha Ali,[d] Nuha Y. Elamin,[b] Faheem Abbas,[e] Abdul Munam Khan,[f] and Rasheed Ahmad Khera *[g]

[a] *Faculty of Materials Science, Shenzhen MSU-BIT University, Shenzhen, 518115, China.*
[b] *Chemistry Department, College of Science, Imam Mohammad Ibn Saud Islamic University (IMSIU), Riyadh 11623, Saudi Arabia.*
[c] *Institute of botany, University of the Punjab, Lahore 54590, Pakistan.*
[d] *Chemical Engineering Research Center, School of Chemical Engineering and Technology, Tianjin University, Tianjin, China.*
[e] *Department of Chemistry, Key Lab of Organic Optoelectronics and Molecular Engineering of Ministry of Education, Tsinghua University, Beijing 100084, PR China.*
[f] *Department of Physics and Microelectronics, Zhengzhou University, Zhengzhou 450001, China.*
[g] *Department of Chemistry, University of Agriculture, Faisalabad 38000, Pakistan.*

Results and Discussion

Mechanical Features

The mechanical performance of $Rb_2AlInCl_6$ and $Rb_2AlInBr_6$ was assessed by computing additional mechanical parameters and analysing their elastic characteristics using elastic constants (ECs). The Born stability criteria are met by both HDPs, confirming their mechanical resilience in cubic crystal structures.[1,2] The bulk modulus (B), which gauges a material's ability to withstand continuous compression, showed significant compressive strength. The result was 22.36 GPa for $Rb_2AlInCl_6$ and 16.45 GPa for $Rb_2AlInBr_6$. Shear modulus (G), which determines opposition to shape distortion, was found to be 7.77 GPa for $Rb_2AlInCl_6$ and 6.54 GPa for $Rb_2AlInBr_6$, suggesting that $Rb_2AlInCl_6$ is relatively stiffer, as depicted **in Fig. S2**.

Young's modulus (E), which measures a substance's strength, was 20.89 GPa for $Rb_2AlInCl_6$ and 17.34 GPa for $Rb_2AlInBr_6$, suggesting that $Rb_2AlInCl_6$ has a higher stiffness against mechanical stretching. For $Rb_2AlInCl_6$ and $Rb_2AlInBr_6$, the Poisson's ratio (v) was found to be 0.34 and 0.32, respectively. $Rb_2AlInCl_6$ seems to be slightly more compressible during transverse deformation because of its higher v. The following relations can be used to calculate below variables:[3–6]

$$G = \frac{1}{2}(G_r + G_v) \tag{1}$$

Where,

$$G_r = \frac{5C_{44}(C_{11} - C_{12})}{3(C_{11} - C_{12}) + 4C_{44}} \text{ and } G_v = \frac{3C_{44} + C_{11} - C_{12}}{5}$$

$$B = \frac{C_{11} + 2C_{12}}{3} \tag{2}$$

$$E = \frac{9GB}{3B + G} \tag{3}$$

$$\upsilon = \frac{3B - 2G}{2(3B + G)} \tag{4}$$

$$A = \frac{2C_{44}}{C_{11} - C_{12}} \tag{5}$$

The Pugh ratio (B/G), a critical indicator of ductility, is computed as 2.87 for $Rb_2AlInCl_6$ and 2.51 for $Rb_2AlInBr_6$. This study demonstrates that both compounds are ductile if the B/G ratio surpasses 1.75. This is additionally corroborated by the Cauchy pressure (C_P) values, which were found to be 7.47 GPa for $Rb_2AlInCl_6$ and 3.45 GPa for $Rb_2AlInBr_6$. Both compounds have ductility when their CP values are positive, whereas $Rb_2AlInCl_6$ exhibits greater ductility.[7,8] $Rb_2AlInCl_6$ and $Rb_2AlInBr_6$ are tested for anisotropy, an indicator for the directional sensitivity of mechanical features,[9–11] with computed values of 0.31 and 0.32. The compared values of mechanical features are listed in **Table S1**. The greater anisotropy value for $Rb_2AlInBr_6$ indicates more elasticity fluctuation depending on the direction of applied stress. Furthermore, the melting temperature (Tm) is calculated using the formula Tm = 553 + 592(C_{12}) ± 300.[12–14] The computed values for $Rb_2AlInCl_6$ and $Rb_2AlInBr_6$ are 804 ± 300K and 750 ± 300K, respectively. Although these values are approximate, the observed decrease from Cl to Br is consistent with the reduction in bond strength and elastic stiffness, indicating weaker lattice cohesion upon halide substitution.

Table S1: The calculation of elastic parameters for Rb_2AlInX_6 (X = Cl, Br)

Parameters	$Rb_2AlInCl_6$	$Rb_2AlInBr_6$	$Rb_2NaCrCl_6$[15]
C_{11}	42.54	33.29	44.59
C_{12}	12.27	8.03	12.09
C_{44}	4.80	4.082	11.61
**B**	22.36	16.45	22.61
**G**	7.77	6.54	13.69
**E**	20.89	17.34	34.18
**A**	0.31	0.32	0.116
**υ**	0.34	0.32	0.248
**B/G**	2.87	2.51	1.65
**Cp**	7.47	3.95	− 0.48
**Tm (K)**	804	750	

Two-dimensional (2D) and three-dimensional (3D) ELATE[16] visual representations, as shown in **Fig. S3** and **Fig. S4,** were developed to better show the mechanical characteristics and computed parameters of $Rb_2AlInCl_6$ and $Rb_2AlInBr_6$. By showing the directional dependency of important elastic parameters, including linear compression, E, G, and *v*, these graphs offer a thorough knowledge of the materials' anisotropic behaviour. These graphs effectively emphasize differences in elastic responses across various crystallographic planes. These examples, which correspond to the computed values and help to understand their mechanical nature, validate the more significant anisotropies in both HDPs. Since both $Rb_2AlInCl_6$ and $Rb_2AlInBr_6$ typically exhibit mechanical stability and ductility, they are both great choices for uses requiring materials with intermediate stiffness and ductility. The ductile behaviour of $Rb_2AlInCl_6$ and $Rb_2AlInBr_6$ depends on the particular application needs. In contrast to $Rb_2AlInBr_6$, $Rb_2AlInCl_6$ exhibits more strength and rigidity.

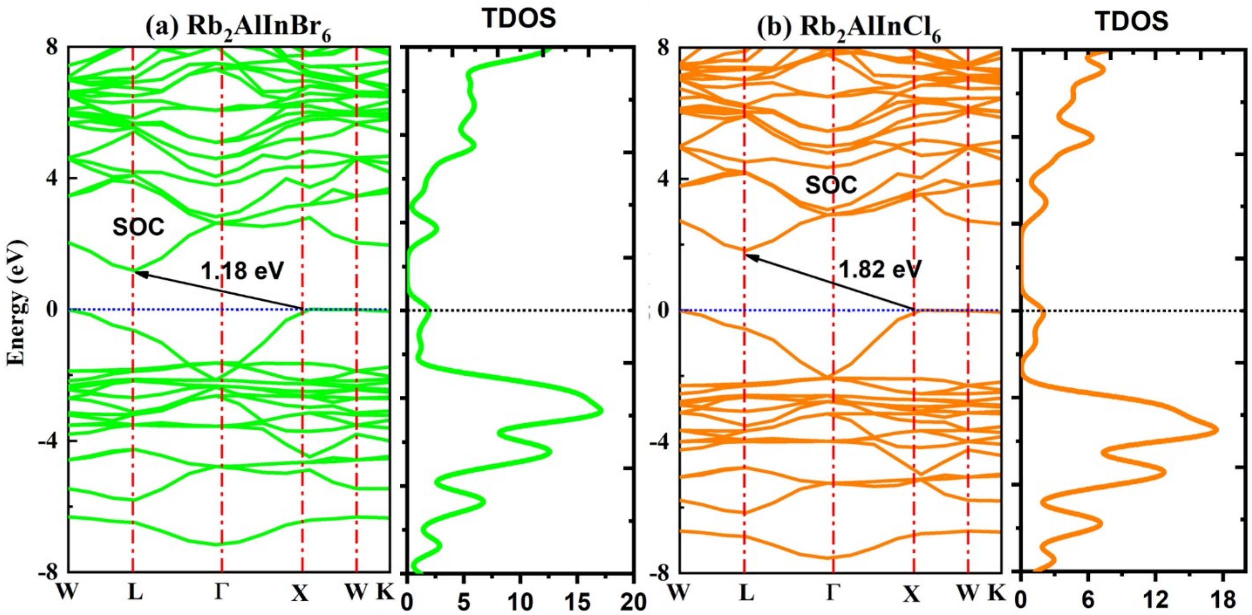

Fig. S1 Band structures and Total density of States for (a) $Rb_2AlInBr_6$ and (b) $Rb_2AlInCl_6$ using SOC

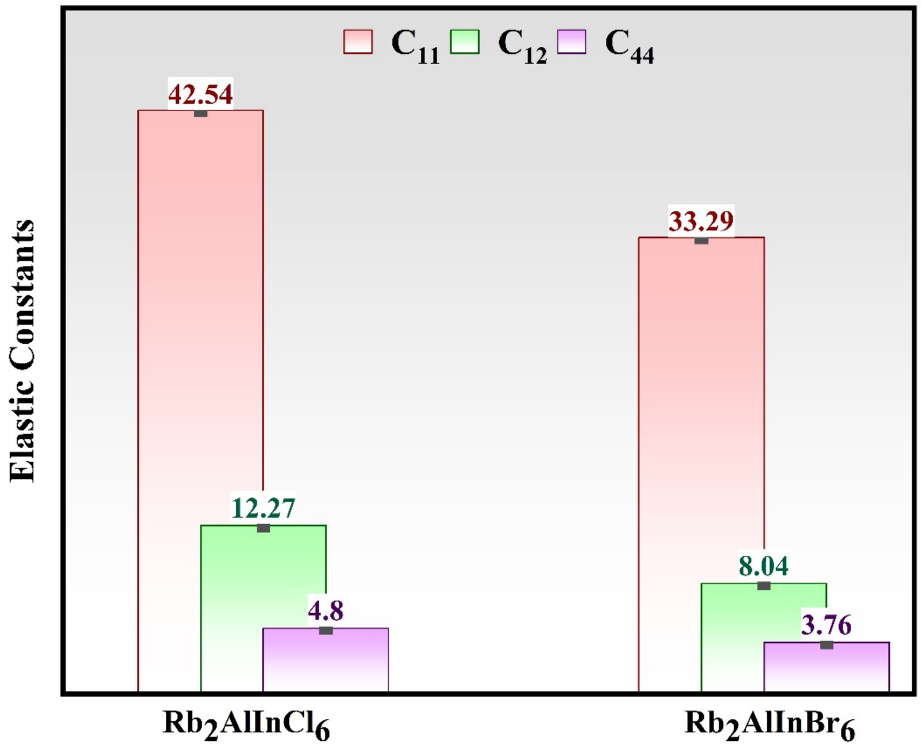

Fig. S2 Elastic Constants of Rb_2AlInX_6 (X = Cl, Br)

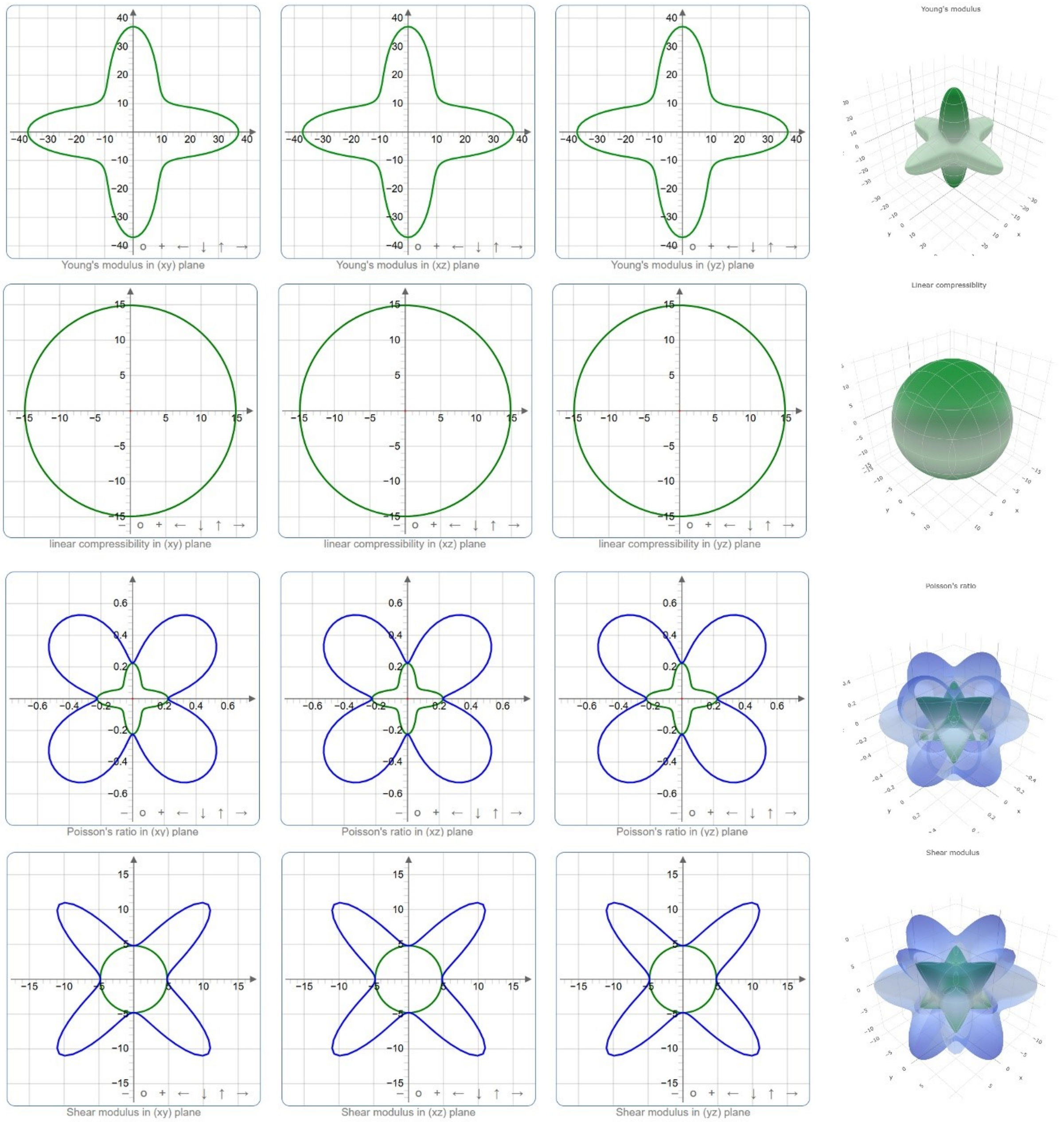

Fig. S3 2D and 3D diagram of (a) G (b) E (c) linear compressibility and (d) v of $Rb_2AlInCl_6$ in different planes

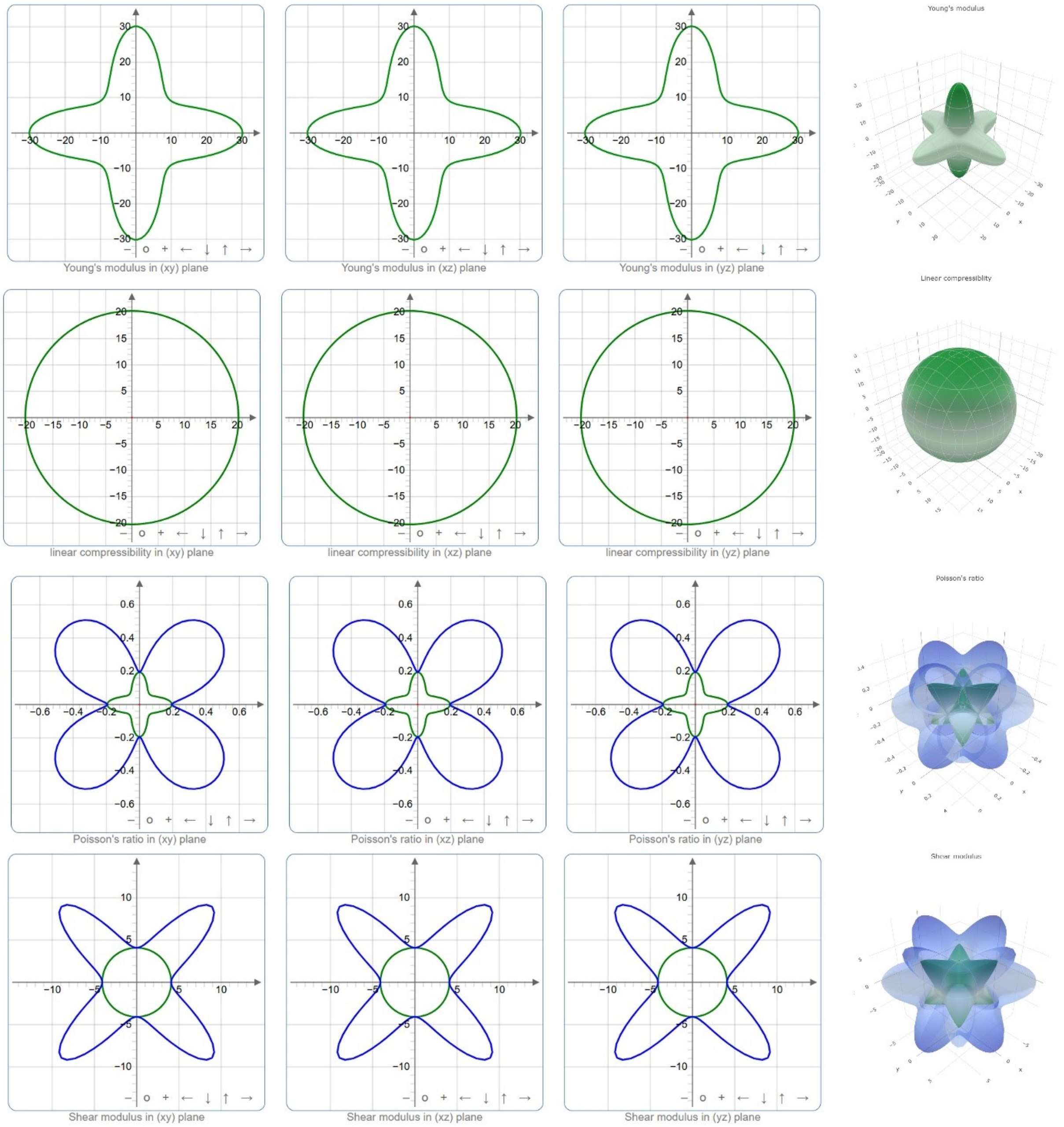

Fig. S4 2D and 3D diagram of (a) G (b) E (c) linear compressibility and (d) v of $Rb_2AlInBr_6$ in different planes

References

1 M. Born, *Mathematical Proceedings of the Cambridge Philosophical Society*, 1940, **36**, 160–172.

2 J. Wang, J. Li, S. Yip, D. Wolf and S. Phillpot, *Physica A*, 1997, **240**, 396–403.

3 S. A. Abbas, M. Rashid, M. A. Faridi, M. B. Saddique, A. Mahmood and S. M. Ramay, *Journal of Physics and Chemistry of Solids*, 2018, **113**, 157–163.

4 Abdullah, N. U. Khan, U. A. Khan, A. Zaman, A. Algahtani, J. Y. Al-Humaidi, V. Tirth, A. M. Alsuhaibani, T. Al-Mughanam, M. S. Refat and A. Ali, *Journal of Physics and Chemistry of Solids*, 2023, **181**, 111479.

5 V. V. Bannikov, I. R. Shein and A. L. Ivanovskii, *physica status solidi (RRL) – Rapid Research Letters*, 2007, **1**, 89–91.

6 M. K. Butt, M. Yaseen, I. A. Bhatti, J. Iqbal, Misbah, A. Murtaza, M. Iqbal, M. mana AL-Anazy, M. H. Alhossainy and A. Laref, *Journal of Materials Research and Technology*, 2020, **9**, 16488–16496.

7 S. Ahmad, J. Feng, M. Zakria, S. Hatim Shah, A. Alam, S. Shakeel, D. Muhammad and I. Ullah, *Materials Science and Engineering: B*, 2024, **309**, 117641.

8 R. Anbarasan, J. K. Sundar, M. Srinivasan and P. Ramasamy, *Computational Condensed Matter*, DOI:10.1016/j.cocom.2021.e00581.

9 V. Tvergaard and J. W. Hutchinson, *Journal of the American Ceramic Society*, 1988, **71**, 157–166.

10 X. Zhang, J. Chen, F. Wang, X. Chen, H. Ma, D. Li, C. Liu and H. Guo, *Ceramics International*, 2019, **45**, 11136–11140.

11 M. Batouche, T. Seddik, S. Uğur, G. Uğur, S. Messekine, T. V. Vu and O. Y. Khyzhun, *Materials Research Express*, DOI:10.1088/2053-1591/ab6254.

12 A. Harbi, A. Aziz and M. Moutaabbid, *Inorganic Chemistry Communications*, 2023, **153**, 110842.

13 S. A. Khandy and D. C. Gupta, *Materials Chemistry and Physics*, 2017, **198**, 380–385.

14 Y. Dhakshayani and G. Kalpana, *Materials Today Communications*, 2025, **42**, 111409.

15 J. Abbas, Y. Xu, K. Riaz, A. Aziz, A. K. Alqorashi and M. Faizan, *Multiscale and Multidiscip. Model. Exp. and Des.*, 2025, **8**, 443.

16 R. Gaillac, P. Pullumbi and F.-X. Coudert, *J. Phys.: Condens. Matter*, 2016, **28**, 275201.
